# Heterogeneity, Characteristics, and Public Health Implications of *Listeria monocytogenes* in Ready-to-Eat Foods and Pasteurized Milk in China

**DOI:** 10.3389/fmicb.2020.00642

**Published:** 2020-04-15

**Authors:** Yuetao Chen, Moutong Chen, Juan Wang, Qingping Wu, Jianheng Cheng, Jumei Zhang, Qifan Sun, Liang Xue, Haiyan Zeng, Tao Lei, Rui Pang, Qinghua Ye, Shi Wu, Shuhong Zhang, Haoming Wu, Wenzhi Li, Xiuying Kou

**Affiliations:** ^1^College of Food Science, South China Agricultural University, Guangzhou, China; ^2^Guangdong Institute of Microbiology, Guangdong Academic of Science, State Key Laboratory of Applied Microbiology Southern China, Guangdong Provincial Key Laboratory of Microbial Culture Collection and Application, Guangdong Open Laboratory of Applied Microbiology, Guangzhou, China; ^3^Infinitus (China) Company, Ltd., Guangzhou, China

**Keywords:** *Listeria monocytogenes*, ready-to-eat foods, LIPI-3, LIPI-4, multi-locus sequence typing, antimicrobial resistance

## Abstract

*Listeria monocytogenes* is a foodborne pathogen with a high mortality rate in humans. This study aimed to identify the pathogenic potential of *L. monocytogenes* isolated from ready-to-eat (RTE) foods and pasteurized milk in China on the basis of its phenotypic and genotypic characteristics. Approximately 7.7% (44/570) samples tested positive for *L. monocytogenes* among 10.8% (39/360) RTE and 2.4% (5/210) pasteurized milk samples, of which 77.3% (34/44) had < 10 MPN/g, 18.2% (8/44) had 10–110 MPN/g, and 4.5% (2/44) had > 110 MPN/g. A total of 48 strains (43 from RTE foods and five from milk samples) of *L. monocytogenes* were isolated from 44 positive samples. PCR-serogroup analysis revealed that the most prevalent serogroup was II.2 (1/2b-3b-7), accounting for 52.1% (25/48) of the total, followed by serogroup I.1 (1/2a-3a) accounting for 33.3% (16/48), serogroup I.2 (1/2c-3c) accounting for 12.5% (6/48), and serogroup II.1 (4b-4d-4e) accounting for 2.1%. All isolates were grouped into 11 sequence types (STs) belonging to 10 clonal complexes (CCs) and one singleton (ST619) via multi-locus sequence typing. The most prevalent ST was ST87 (29.2%), followed by ST8 (22.9%), and ST9 (12.5%). Virulence genes determination showed that all isolates harbored eight virulence genes belonging to *Listeria* pathogenicity islands 1 (LIPI-1) (*prfA*, *actA*, *hly*, *mpl*, *plcA*, *plcB*, and *iap*) and *inlB*. Approximately 85.4% isolates carried full-length *inlA*, whereas seven isolates had premature stop codons in *inlA*, six of which belonged to ST9 and one to ST5. Furthermore, LLS (encoded by *llsX* gene, representing LIPI-3) displays bactericidal activity and modifies the host microbiota during infection. LIPI-4 enhances neural and placental tropisms of *L. monocytogenes*. Results showed that six (12.5%) isolates harbored the *llsX* gene, and they belonged to ST1/CC1, ST3/CC3, and ST619. Approximately 31.3% (15/48) isolates (belonging to ST87/CC87 and ST619) harbored *ptsA* (representing LIPI-4), indicating the potential risk of this pathogen. Antimicrobial susceptibility tests revealed that > 95% isolates were susceptible to 16 antimicrobials; however, 60.4 and 22.9% isolates were intermediately resistant to streptomycin and ciprofloxacin, respectively. The results show that several isolates harbor LIPI-3 and LIPI-4 genes, which may be a possible transmission route for *Listeria* infections in consumers.

## Introduction

*Listeria monocytogenes* is a foodborne pathogen with a high mortality rate in humans. The vulnerable groups, such as the elderly, pregnant women, fetuses and immunocompromised individuals are the main population infected by *L. monocytogenes* (Cartwright et al., 2013). This pathogen has high tolerance against stressors associated with food processing, including refrigerating temperatures, low moisture content, high salinity, and a wide pH range (Hain et al., 2007). Furthermore, sequence type (ST) 8, ST5, ST87, ST2, ST121, ST14, and ST9 subtypes of *L. monocytogenes* are present and grow in some specific niches, resulting in long-term contamination during food production and processing (Fagerlund et al., 2016; Chen et al., 2017; Chau et al., 2017; Pasquali et al., 2018; Melero et al., 2019).

*Listeria* infections potentially result from ingestion of *L. monocytogenes*- contaminated foods. In 2017–2018, the largest listeriosis outbreak worldwide occurred in South Africa, resulting from ingestion of ready-to-eat (RTE) meat contaminated with *L. monocytogenes* ST6 (Smith et al., 2019). Previous studies reported an incidence of 0.46 cases per 100,000 individuals in Europe in 2015 (European Food Safety Authority and European Centre for Disease Prevention and Control, 2016). According to previous study, 147 clinical cases, 479 *Listeria* isolates and 82 outbreak-related cases were reported from 28 provinces between 1964 and 2010 in China, respectively (Feng et al., 2013). Fan et al. (2019) conducted a systematic review for the listeriosis in mainland China, wherein 136 records were identified, and reporting 562 patients with listeriosis from 2011 to 2017, indicating a drastic increase in the number of patients over the past decade. Listeriosis patients were primarily reported in developed cities (Beijing city and the coastal cities), probably owing to dietary habits and the high population density in these areas. Risk identification of *L. monocytogenes* in RTE foods is important because they provide critical information regarding food sources of listeriosis. Therefore, comprehensive surveillance of *L. monocytogenes* in RTE foods throughout China is of utmost importance.

Thirteen serotypes of *L. monocytogenes* have been assigned based on the somatic (O) and flagellar (H) antigens. Noticeably, serotypes 4b, 1/2a, 1/2b, and 1/2c account for > 95% of isolates recovered from foods and clinical cases (Pontello et al., 2012). At present, multi-locus sequence typing (MLST) (Ragon et al., 2008), and core genome MLST (cgMLST) (Chen et al., 2016) are used for molecular typing of *L. monocytogenes*. MLST is a reliable, high-resolution, and acceptable method for typing *L. monocytogenes*. Furthermore, the molecular virulence of *L. monocytogenes* isolates is closely related to the distribution patterns of virulence genes of *Listeria* pathogenicity island-1 (LIPI-1) and the *inlAB* operon in the genome (Vazquez-Boland et al., 2001). LIPI-1 includes *prfA*, *actA*, *hly*, *mpl*, *iap*, *plcA*, *plcB* genes (Chen et al., 2018a) and the *inlAB* operon encodes two internalins (InlA and InlB), which are critical for entry into hepatocytes (Gaillard et al., 1996). PCR analysis indicated that majority of *L. monocytogenes* isolates contained most of virulence genes of LIPI-1 and *inlAB* operon (Chen et al., 2018a, 2019a; Basha et al., 2019; Zhang et al., 2019). Additional lineage and STs specific pathogenicity islands, for example, *llsX* gene in LIPI-3 encodes the bacteriocin Listeriolysin S (LLS), is associated with hemolytic and cytotoxic activity, displays bactericidal activity and modifies the host microbiota during infection (Cotter et al., 2008; Quereda et al., 2017; Radoshevich and Cossart, 2018; Yin et al., 2019); LIPI-4, a putative cellobiose-family phosphotransferase system, is responsible for the neural and placental tropisms infection of *L. monocytogenes*, respectively (Maury et al., 2016). Previous studies reported *llsX* gene from LIPI-3 present in ST1, ST3, ST4, ST6, ST77, ST79, ST213, ST217, ST224, ST288, ST308, ST323, ST330, ST363, ST380, ST382, ST389, ST489, ST554, ST581, ST619, ST778, ST999, ST1000 and ST1001, and *pstA* gene from LIPI-4 present in ST4, ST87, ST213, ST217, ST310, ST363, ST382, ST619, ST663, ST1002, and ST1166 have been reported (Chen et al., 2018a, b, 2019b; Kim et al., 2018; Wang et al., 2018). Therefore, the virulence of different ST strains of *L. monocytogenes* may vary.

This study aimed to detect and enumerate *L. monocytogenes* in RTE foods and pasteurized milk products and to determine their heterogeneity, characteristics, and public health implications in Chinese retail outlets.

## Materials and Methods

### Samples

Between March 2014 and June 2016, 360 RTE foods and 210 pasteurized milk samples were collected from 21 cities in China, including cold vegetable dish in sauce (*n* = 60 samples), duck (*n* = 77), fried rice (*n* = 15), chicken (*n* = 139), pork (*n* = 61), goat meat (*n* = 8), and pasteurized milk (*n* = 210). All of RTE foods were loose-packed in different markets. All samples were immediately placed in sterile bags, kept in an insulated shipping cooler with frozen gel packs placed on the sides, middle, and above the samples to maintain below 4°C. All the samples were transferred back to the laboratory immediately and analyzed within 4 h of receiving the samples.

### Qualitative and Quantitative Analysis

Qualitative detection of *L. monocytogenes* was performed on the basis of the guidelines of the National Food Safety Standard of China (4789.30-2010) (Anonymous, 2010), with minor modifications. Briefly, 25 g (mL) of homogenized samples were added to 225 mL *Listeria* enrichment broth 1 (LB1) (Guangdong Huankai, Co. Ltd., Guangzhou, China). The cultures in LB1 media were incubated at 30°C for 24 h. After incubation, 100 μL of the LB1 enrichment culture was transferred to 10 mL *Listeria* enrichment broth 2 (LB2) (Guangdong Huankai, Co. Ltd.) and incubated at 30°C for 24 h. A loopful (about 10 μL) of the LB2 enrichment culture was streaked onto *Listeria* CHROMagar plates (CHROM-agar, Paris, France) and incubated at 37°C for 48 h. At least three (when possible) presumptive colonies were selected for the identification of *L. monocytogenes*, using the Microgen ID *Listeria* identification system (Microgen, Camberley, United Kingdom) in accordance with the manufacturer’s instructions.

For quantitative detection, the most probable number (MPN) method using a nine-tube was followed, as reported previously (Gombas et al., 2003). Briefly, nine tubes were divided into three sets of three tubes each. Homogenized samples (25 g) were added to 225 mL half Fraser Broth (Guangdong Huankai, Co. Ltd.). The first set of tubes contained 10 mL of the sample homogenate in 225 mL half Fraser Broth, while the second and third sets contained 10 mL of half Fraser Broth inoculated with 1 and 0.1 mL of the homogenate, respectively. Different volumes, i.e., 10, 1, and 0.1 mL, of the sample homogenate represented 1.0, 0.1, and 0.01 g of the original sample, respectively. The nine tubes were incubated at 30 ± 2°C for 24 ± 2 h. The darkened Fraser tubes were streaked onto *Listeria* CHROMagar plates. If a Fraser Broth tube did not display darkening, it was reexamined after an additional 26 ± 2 h of incubation. The presumptive colonies were purified again on the Tryptic Soy Agar (TSA) plates (Guangdong Huankai, Co. Ltd.) and then identified using the Microgen ID *Listeria* identification system. The MPN value was calculated based on the number of positive tube(s) in each sample and the MPN table (U.S. Department of Agriculture, 1998).

### PCR-Serogroup Analysis and Virulence Genes Determination

Genomic DNA was extracted from *L. monocytogenes* isolates using a HiPure Bacterial DNA Kit (Guangzhou Magen Biotechnology, Co. Ltd., Guangzhou, China) according to the manufacturer’s instructions. Serogroups of the *L. monocytogenes* isolates were differentiated via a multiplex PCR method reported previously by Doumith et al. (2004) (Supplementary Table S1). PCR was carried out using a thermal cycler (Biometra, Göttingen, Germany) at the following conditions: initial denaturation at 94°C for 3 min; followed by 35 cycles at 94°C for 35 s, 53°C for 50 s, and 72°C for 60 s, and final extension at 72°C for 5 min. The isolates were determined as serogroup I.1 (1/2a-3a), I.2 (1/2c-3c), II.1 (4b-4d-4e), II.2 (1/2b-3b-7), and III (4a-4c).

Virulence genes including LIPI-1 (*prfA*, *actA*, *hly*, *mpl*, *plcA*, *plcB*, *iap*) and *inlA*, *inlB* were detected via PCR. Furthermore, two additional PCRs were carried out to detect the *llsX* and *ptsA* genes (representing LIPI-3 and LIPI-4, respectively) in the *L. monocytogenes* isolates (Clayton et al., 2011; Maury et al., 2016). The PCR primers used herein are enlisted in Supplementary Table S1. The amplicons were separated on 1.5% agarose gels in TAE buffer (Biosharp, Co., Ltd., Hefei, China) and visualized via Goldview^®^ (Beijing Solarbio Science & Technology, Co., Ltd., China) staining (0.005%, v/v). To determine the premature stop codon (PMSC) in the *inlA* gene, full-length *inlA* of each isolate was amplified and sequenced using external and internal primers (Wu et al., 2016). Compared to previous reported PMSCs types (Chen et al., 2018a), the PMSCs in the *inlA* gene were analyzed and determined using MEGA X software (Kumar et al., 2018).

### Antimicrobial Susceptibility Test

The antimicrobial susceptibility of the *L. monocytogenes* isolates was assessed using the disk diffusion method based on breakpoints for *Staphylococci* spp. in accordance with the guidelines of the Clinical Laboratory Standards Institute (Clinical and Laboratory Standard Institute, 2017). The breakpoints of ampicillin and penicillin G for specific *Listeria* spp. have been defined (M45-A2 Vol. 30 No. 18). The following 17 common antimicrobial agents (disk load), including those used to treat human listeriosis, were assessed herein: kanamycin (30 μg), gentamicin (10 μg), ciprofloxacin (5 μg), levofloxacin (5 μg), ofloxacin (5 μg), sulfamethoxazole with trimethoprim (23.75/1.25 μg), streptomycin (10 μg), rifampin (5 μg), doxycycline (30 μg), chloramphenicol (30 μg), erythromycin (15 μg), tetracycline (30 μg), meropenem (10 μg), vancomycin (30 μg), linezolid (30 μg), amoxycillin/clavulanic acid (10 μg), and sulbactam/ampicillin (10/10 μg) (Oxoid, Basingstoke, United Kingdom). Briefly, *L. monocytogenes* isolates were seeded in tryptone soya broth supplemented with 0.6% yeast extract (TSB-YE) (Guangdong Huankai, Co. Ltd.) and incubated at 37°C overnight. The cell density of the suspension was adjusted to 1.0 McFarland standard, which is approximately 3 × 10^8^ CFU/mL, and then diluted to ∼10^5^ CFU/mL with 0.85% NaCl (w/v). The suspension was spread onto the surface of Mueller-Hinton agar (Guangdong Huankai, Co. Ltd.). The diameters of the inhibition zones were measured using precision calipers after 24 h of incubation. *Staphylococcus aureus* ATCC 25923 and *Escherichia coli* ATCC 25922 were used as quality control strains. Multidrug-resistant isolates were defined as isolates displaying resistance to at least three classes of antimicrobial agents assessed herein (Magiorakos et al., 2012).

### MLST Analysis

Using the method of Ragon et al. (2008), MLST analysis of *L. monocytogenes* was performed on the basis of the following seven housekeeping genes: *acbZ* (ABC transporter), *bglA* (beta-glucosidase), *cat* (catalase), *dapE* (Succinyl diaminopimelate desuccinylase), *dat* (D-amino acid aminotransferase), *ldh* (lactate dehydrogenase), and *lhkA* (histidine kinase) (Supplementary Table S2). A detailed protocol for the present MLST analysis, including primers, PCR conditions, was in accordance with the guidelines of the Pasteur Institute website^1^, and STs and clonal complexes (CCs) of each isolate were assigned on the basis of each variant locus of each housekeeping gene. A phylogenetic tree was generated to analyze relationships among the isolates, using MEGA X (Kumar et al., 2018).

## Results

### Occurrence and Contamination Levels of *L. monocytogenes*

As shown in Table 1, 7.7% samples (39 RTE foods and 5 pasteurized milk samples) tested positive for *L. monocytogenes* from among 570 food samples. The contamination rate of goat meat (25.0%, 2/8) was the highest among the 7 types of RTE foods and milk, followed by fried rice (20.0%, 3/15) and a cold vegetable dish in sauce (15.0%, 9/60), chicken (11.5%, 16/139), duck (7.8%, 6/77), pork (4.9%, 3/61), and pasteurized milk (2.4%, 5/210). For quantitative analysis, 77.3% (34/44) samples contaminated with *L. monocytogenes* were below 10 MPN/g, 18.2% (8/44) were between 10 and 110 MPN/g, and only two (4.5%) samples were over 110 MPN/g, constituting sesame oil chicken and pasteurized milk, respectively.

**TABLE 1:** Contamination levels of *Listeria monocytogenes* in ready-to-eat foods and pasteurized milk.

Samples	0.3 ≤ MPN < 10	10 ≤ MPN < 110	≥110	Contamination rate
Cold vegetable	8	1	0	15.0%(9/60)
dish in sauce				
Duck	4	2	0	7.8%(6/77)
Fried rice	2	1	0	20.0%(3/15)
Chicken	12	3	1	11.5%(16/139)
Pork	2	1	0	4.9%(3/61)
Goat meat	2	0	0	25.0%(2/8)
Milk	4	0	1	2.4%(5/210)
Total	77.3% (34/44)	18.2% (8/44)	4.5% (2/44)	7.7%(44/570)

### PCR-Serogroup Analysis

**FIGURE 1:**
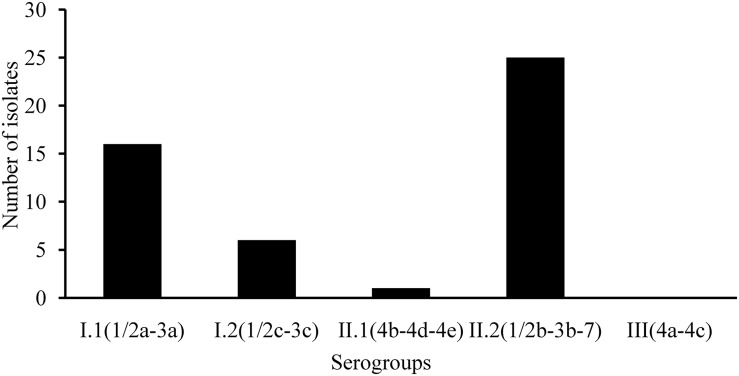
Serogroup identification of *Listeria monocytogenes* isolated from ready-to-eat foods and pasteurized milk.

The ERIC-PCR fingerprinting was used to screen isolates from the same sample in order to remove the duplicate isolates (data not shown). *L. monocytogenes* isolates from the same sample with > 90% similarity was considered as clonal. Clonal isolates from individual sample were excluded. At least one *L. monocytogenes* isolate from each known source sample was submitted to further analysis. A total of 48 strains of *L. monocytogenes* were isolated from 44 *L. monocytogenes*-positive samples, including four samples (Cold vegetable dish in sauce, Pickled pig ear, Roasted chicken wing, and Salt baked chicken), each containing two different isolates. *L. monocytogenes* isolates recovered from RTE foods (43 isolates) and pasteurized milk samples (five isolates) were subjected to serogroup assessment via multiplex PCR analysis. As shown in Figure 1, 33.3% (16/48) isolates belonged to serogroup I.1 (1/2a-3a); 12.5% (6/48), serogroup I.2 (1/2c-3c); 2.1% (1/48), serogroup II.1 (4b-4d-4e); 52.1% (25/48), serogroup II.2 (1/2b-3b-7). None of the isolates belonged to serogroup III (4a-4c).

### Antimicrobial Susceptibility Test

**FIGURE 2:**
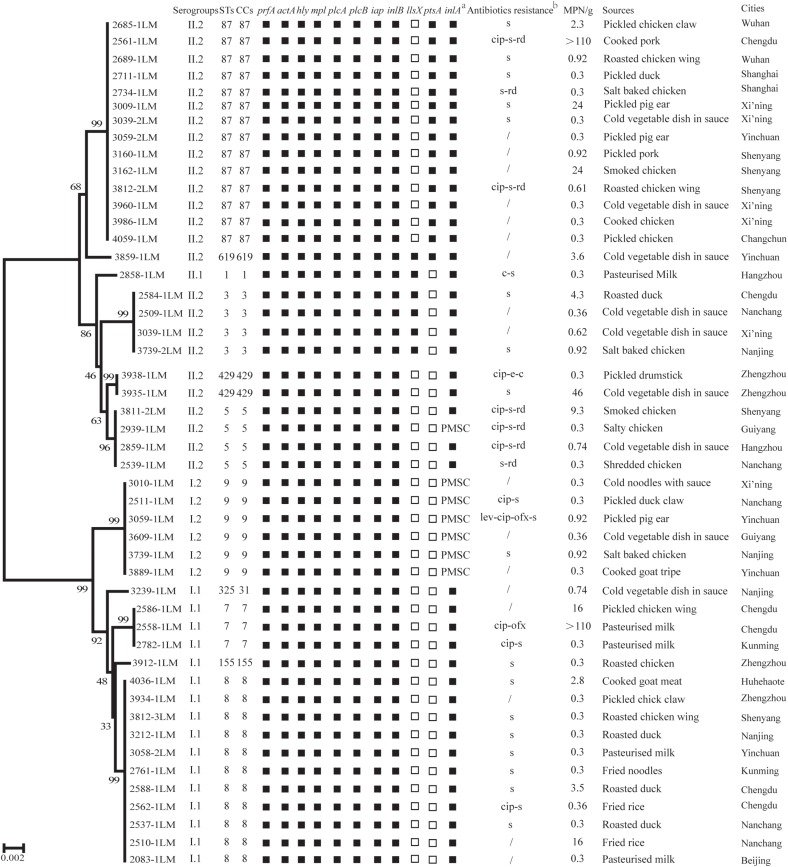
The phenotypic and genotypic characteristics of *Listeria monocytogenes* isolates. Of the 10 virulence genes (*prfA*, *actA*, *hly*, *mpl*, *plcA*, *plcB*, *iap*, *inlB*, *llsX*, and *ptsA*), black squares indicate the presence of the corresponding gene; white squares represent lack of corresponding gene. (a) PMSC, premature stop codons in *inlA*; black squares indicate the presence of full-length *inlA*. (b) c, chloramphenicol; cip, ciprofloxacin; e, erythromycin; lev, levofloxacin; ofx, ofloxacin; rd, rifampin; s, streptomycin;/indicates no resistance. Antibiotic abbreviations in lowercase indicate intermediate resistance. A neighbor-joining tree of *L. monocytogenes* based on the MLST of seven housekeeping genes was established using MEGA X with 1000 bootstrap replications. Bootstrap values are shown at the nodes.

Susceptibility toward 19 antimicrobials was assessed among the 48 *L. monocytogenes* isolates. As shown in Supplementary Table S3, all isolates were susceptible to 12 antimicrobials, including kanamycin, gentamicin, sulfamethoxazole with trimethoprim, doxycycline, tetracycline, meropenem, vancomycin, linezolid, amoxicillin/clavulanic acid, sulbactam/ampicillin, ampicillin, and penicillin. More than 95.0% of isolates were susceptible to four antimicrobials (levofloxacin, ofloxacin, chloramphenicol, and erythromycin). However, 60.4% (29/48), 22.9% (11/48), and 14.6% (7/48) isolates were intermediately resistant to streptomycin, ciprofloxacin and rifampin, respectively. Seven resistance profiles were obtained for 48 *L. monocytogene*s isolates (Figure 2).

### MLST Analysis and Virulent Genes Profiles

**FIGURE 3:**
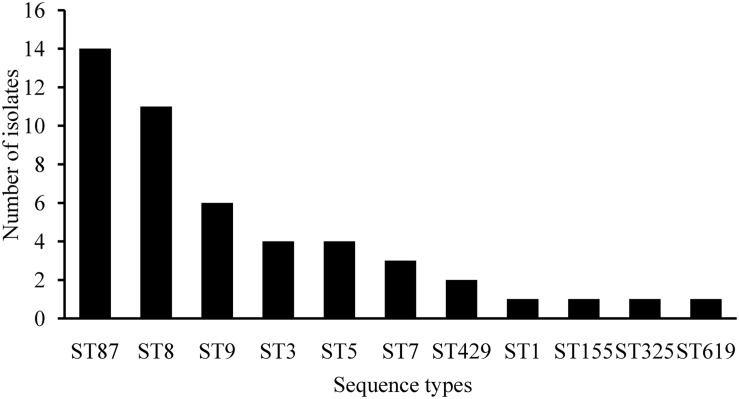
The sequence type distributions of *Listeria monocytogenes* isolated from ready-to-eat foods and pasteurized milk.

The genetic diversity of 48 isolates obtained from RTE foods and pasteurized milk were subjected to MLST analysis. As shown in Figure 3, 48 isolates belonged to 11 STs. ST87/CC87 (29.2%, 14/48) displayed the highest prevalence, followed by ST8/CC8 (22.9%, 11/48) and ST9/CC9 (12.5%, 6/48). The remaining eight CCs displayed a scattered distribution: ST3/CC3 (four isolates), ST5/CC5 (four isolates), ST7/CC7 (three isolates) ST429/CC429 (two isolates), ST1/CC1 (one isolates), ST155/CC155 (one isolates), ST325/CC31 (one isolates), and ST619 (one isolates). Virulence genes including LIPI-1, *inlA*, and *inlB* were present in all 11 STs/CCs isolates, while *llsX* (representing LIPI-3) was present exclusively in ST619 (one isolate), ST1/CC1 (one isolate), and ST3/CC3 (four isolates); ST619 (one isolate) and ST87/CC87 (14 isolates) isolates harbored the *ptsA* gene (representing LIPI-4). As shown in Figure 2, six isolates belonged to ST9/CC9 (6/6, 100%) and one isolate belonged to ST5/CC5 (1/4, 25%) and harbored PMSCs in the *inlA* gene. An adenylic acid deletion occurred at position 12 in the *inlA* gene in three isolates (2739-1LM, 3010-1LM, and 2511-1LM). A nonsense mutation at position 978 (GAA→TAA) in isolate 3609-1LM, position 1380 (TGG→TGA) in isolate 3883-1LM, and position 1605 (TGG→TGA) in isolate 2939-1LM were identified. In isolate 3059-1LM, a base of adenylic acid deletion occurred at position 1637, resulting in PMSCs in the *inlA* gene.

## Discussion

*Listeria monocytogenes* is a prominent facultative foodborne pathogen with a worldwide prevalence. Numerous listeriosis outbreaks and sporadic cases have resulted from ingestion of *L. monocytogenes*-contaminated foods. In particular, RTE foods are considered vehicles of *Listeria* spp. owing to the absence of further heat treatment before consumption. The occurrence of listeriosis varies among different countries and usually occurs at 0.1–11.3 cases per million individuals (FAO/WHO, 2004). In this study, we assessed 570 food samples in China from 2014 to 2016, 39 RTE foods and 5 pasteurized milk samples (7.7%) tested positive for *L. monocytogenes*, concurrent with contamination of *L. monocytogene*s in RTE foods reported in Beijing (Wang W. et al., 2015) but significantly greater than that of RTE foods in Estonia (Koskar et al., 2019) and Tokyo, Japan (Shimojima et al., 2016). However, Wang et al. (2018) reported that 16.2% of samples were contaminated with *L. monocytogenes* in retail outlets selling RTE foods and restaurants serving mutton in Zigong, Sichuan Province. Among the pasteurized milk samples, only 2.4% (5/210) samples were positive for *L. monocytogenes*. Nonetheless, the contamination rate in other countries is markedly greater than that reported herein in China, amounting to 20% among pasteurized milk in Ethiopia (Seyoum et al., 2015) and 4.9% in milk and milk products in Tamil Nadu, India (Karthikeyan et al., 2015). This discrepancy is potentially attributed to differences in the sample size, sample constitution, hygiene conditions, or geographical locations.

Risk assessment of a *L. monocytogenes* infection through ingestion of RTE foods depended not only on the contamination rate of RTE foods, but also on their level of contamination. To limit the occurrence of listeriosis, several countries have formulated a series of food microbiological standards for *L. monocytogenes* for different foods. The United States Department of Agriculture (USDA) has adopted a zero-tolerance policy (absence of the organism in 25 g food sample) for *L. monocytogenes* in RTE foods (Orsi et al., 2011). The EU food safety regulations limit *L. monocytogenes* to < 100 CFU/g at the end of the shelf-life of a food product (Ziegler et al., 2019). Furthermore, China has adopted a zero-tolerance policy for RTE meat products (GB 29921-2013) (Anonymous, 2013); however, such regulations have not been adopted for pasteurized milk. In many European countries, *L. monocytogenes* counts must do not exceed 100 CFU/g during the shelf-life of RTE foods (Castro et al., 2017). Herein, 77.3% (34/44) samples were contaminated with *L. monocytogenes* below 10 MPN/g, 18.2% (8/44) were between 10 and 110 MPN/g, and only two (4.5%) samples were over 110 MPN/g. Concurrently, Koskar et al. (2019) reported that 0.3% of positive samples exceeded the food safety criterion of 100 CFU/g. The food matrix, storage time, and temperature were significant factors influencing the growth of *L. monocytogenes* (Ziegler et al., 2019). Results showed that the most common STs were ST8 strains in fried rice/noodles (100%, 3/3) and duck (50%, 3/6) isolates, while the ST87 strains (carried *ptsA* gene) were most found in chicken (44.4%, 8/18) and pork (75%, 3/4) isolates. The contamination rate of positive samples collected from Nanchang and Chengdu city were the highest among 43 cities, both being 50% (5/10), followed by Xi’ning (25%, 5/20). The count of *L. monocytogenes* in these positive samples may have drastically increased owing to the presence of numerous nutrients and an ambient storage temperature. The present results indicate that RTE foods are potentially an important transmission route for *L. monocytogenes* infections. Further surveillance of *L. monocytogenes* in RTE foods is warranted for risk assessment.

Serotyping is a classical typing method for *L. monocytogenes.* Serotypes 1/2a, 1/2b, 4b, and 1/2c account for 95% of clinical isolates (Pontello et al., 2012; Silk et al., 2012). In this study, 33.3% (16/48) isolates belonged to serogroup I.1 (1/2a-3a); 12.5% (6/48), serogroup I.2 (1/2c-3c); 2.1% (1/48), serogroup II.1 (4b-4d-4e); 52.1% (25/48), serogroup II.2 (1/2b-3b-7); this was concurrent with previous reports from China (Chen et al., 2009, 2015). For MLST analysis, 48 isolates belonging to 11 STs, ST87/CC87 (29.2%, 14/48), displayed the highest prevalence, followed by ST8/CC8 (22.9%, 11/48) and ST9/CC9 (12.5%, 6/48). Interestingly, ST87/CC87 was dominant in the listeriosis cases in China (Wang Y. et al., 2015). Huang et al. (2015) reported that ST87/CC87 were predominant among the 132 isolates obtained from listeriosis cases during 2000–2013 in Taiwan Province, China. Wang et al. (2018) reported that 24.2% (8/33) of listeriosis cases resulted from CC87 in Zigong, Sichuan Province; similarly, clinical cases resulted from RTE foods contaminated with ST87/CC87 strains (Zhang et al., 2018). These data suggest that ST87 poses potential health risks to consumers. To date, ST87/CC87-associated outbreaks and sporadic cases were seldom reported in other countries. However, 27 human listeriosis outbreaks primarily resulted from ST87/CC87 strains (One isolated from the foie gras that the patient ate) in Spain in 2013–2014 (Perez-Trallero et al., 2014), indicating that ST87/CC87 may potentially emerge as a hypervirulent strain worldwide. Furthermore, Chau et al. (2017) reported that ST87 was present in a particular brand of pre-packed salmon products over a 4-year period, implying a potential persistent contamination issue at the level of production. The predominance of ST87/CC87 of *L. monocytogenes* in foods is potentially associated with listeriosis, and further analysis of genotypic characteristics of foodborne and clinical isolates is required.

Virulence genes (LIPI-1, *inlA*, and *inlB*) were present in all *L. monocytogenes* isolates. Internalin A (InlA, encoded by *inlA*) is a major virulence factor associated with the invasiveness of *L. monocytogenes* and binds E-cadherin on host cells and facilitates the penetration of *L. monocytogenes* into intestinal epithelial cells (Vazquez-Boland et al., 2001). Therefore, PMSCs in the *inlA* gene may attenuate the virulence of *L. monocytogenes* strains with a lesser invasive phenotype among human intestinal epithelial cells (Nightingale et al., 2005; Van Stelten and Nightingale, 2008; Van Stelten et al., 2010). Interestingly, six (100%) ST9/CC9 and one (25%) ST5/CC5 isolates harbored PMSCs in the *inlA* gene, concurrent with previous studies reported in China (Chen et al., 2018a, 2019a). Similarly, the PMSCs in the *inlA* gene were frequent in ST121/CC121 strains, which were predominant in foods and associated environments (Ciolacu et al., 2015; Palma et al., 2017; Rychli et al., 2017, 2018). These data indicate that the ST9 and ST121 strains predominant in foods may acquire PMSCs in the *inlA* gene. However, the ecological significance of specific InlA variants is not yet understood (Ciolacu et al., 2015). Further research should be conducted to elucidate whether the PMSCs of *inlA* is associated with food-related stress. The bacteriocin LLS (encoded by the *llsX* and other genes) contributes to intestinal survival and virulence of *L. monocytogenes* in a murine oral infection model, alters the gut microbiota and increases *L. monocytogenes* persistence (Quereda et al., 2016). ST1, ST3, and ST619 strains harbored LIPI-3 (representing *llsX*), consistent with these STs also causing listeriosis both in China and other countries (Lv et al., 2014; Maury et al., 2016; Wang et al., 2018). ST87/CC87 (and ST619) strains harbored LIPI-4 (representing *ptsA*), responsible for neural and placental infections (Maury et al., 2016). It is noteworthy that one ST619 isolate had all virulence genes tested (especially *llsX* and *ptsA*), indicating a potential hypervirulent ST. Multiple reports also shown that ST619 strain was isolated in food and carried many virulence genes, including *llsX* and *ptsA* genes (Chen et al., 2018a, b, 2019b; Wang et al., 2018). In addition, to the best our knowledge, ST619 was only reported in clinic cases in China (Wang et al., 2018). However, to date, little information is available on the pathogenicity of ST619 strains, it is necessary to explore the virulence of ST619 strains in the future. Our data suggest that RTE foods and pasteurized milk contaminated with the isolates recovered herein may increase the risk of *L. monocytogenes* infection for consumers in China.

Listeriosis may be more difficult to control in future owing to the emergence of antimicrobial resistance among *L. monocytogenes* strains isolated from food products. β-lactam (penicillin and ampicillin) alone or in combination with an aminoglycoside (gentamycin) has been the first-line treatment alternative for listeriosis. However, patients presenting an allergic reaction to penicillin, a second-line treatment alternative, usually involve a combination of trimethoprim with a sulfonamide including sulfamethoxazole in co-trimoxazole (Alonso-Hernando et al., 2012). Fortunately, all 48 *L. monocytogenes* isolates were susceptible to β-lactam and aminoglycoside antimicrobials herein, indicating that these antimicrobials are still effective to treat listeriosis. Furthermore, rifampicin, tetracycline, chloramphenicol, and fluoroquinolones have been used to treat listeriosis (Allerberger and Wagner, 2010). Herein, *L. monocytogenes* isolates were intermediately resistant, to a certain extent, to fluoroquinolones, although no completely resistant isolate was observed. The mechanisms underlying fluoroquinolones resistance have been previously reported. Fluoroquinolone resistance may be mediated by mutations in quinolone resistance-determining regions, including GyrA, GyrB, ParC, and ParE (Bertrand et al., 2016). In addition, the expression of efflux pump FepR, MdeL, and Lde contributed to resistance toward fluoroquinolones (Lismond et al., 2008; Guerin et al., 2014; Lim et al., 2016; Wilson et al., 2018). Vancomycin and novel β-lactams constitute last-line therapy for *Listeria* infections, and no resistant isolate was observed herein. Therefore, future studies are required to focus on the mechanism underlying the acquisition of fluoroquinolone resistance in *L. monocytogenes*.

## Conclusion

In summary, 7.7% (44/570) of RTE foods (39/360) and pasteurized milk (5/210) samples collected from 21 cities in China were positive for *L. monocytogenes* and two samples had a MPN > 110/g. Serogroup I.1 (ST8, 22.9%) and II.2 (ST87, 29.2%) were dominant among the 48 *L. monocytogenes* isolates, indicating that some specific serogroups and STs of *L. monocytogenes* may have distinct ecological niches. The nine classical virulence genes and additional *llsX* or/and *ptsA* potential hypervirulent genes were present in some specific *L. monocytogenes* isolates, indicating that the *L. monocytogenes* contaminated RTE foods and pasteurized milk may be a possible transmission route for *Listeria* infection in consumers. Although most isolates were susceptible to antimicrobials, except for streptomycin and ciprofloxacin (efflux pump mediated resistance), potential microbial safety issues in RTE foods and pasteurized milk requires close attention.

## Data Availability Statement

The raw data supporting the conclusions of this manuscript will be made available by the authors, without undue reservation, to any qualified researcher.

## Author Contributions

QW, JZ, and MC conceived and designed the experiments. MC, YC, JC, and QS performed the experiments. LX, HZ, SW, RP, and HW conducted the bioinformatics analyses. MC, QW, SZ, and TL drafted the manuscript. QW, QY, JW, WL, and XK reviewed the manuscript. All authors read and approved the final manuscript.

## Conflict of Interest

WL and XK were employed by Infinitus (China) Company, Ltd. The remaining authors declare that the research was conducted in the absence of any commercial or financial relationships that could be construed as a potential conflict of interest.

## Footnotes

1 http://bigsdb.pasteur.fr/listeria/listeria.html
