# Supplementary material for: Heterogeneity, Characteristics, and Public Health Implications of Listeria monocytogenes in Ready-to-Eat Foods and Pasteurized Milk in China
Source: Front Microbiol. 2020 Apr 15;11:642. doi: 10.3389/fmicb.2020.00642 (PMC7174501; doi:10.3389/fmicb.2020.00642)
Supplement: Supplementary file 1 [file Table_1.docx]

Table S1 Primers used for serogroups and virulence genes identification of *Listeria monocytogenes* strains

| Target gene | Forward and reverse primers (5'→3') | Specificity | Tm (°C) | Size of PCR amplicon (bp) | Reference |
| --- | --- | --- | --- | --- | --- |
| *lmo0737* | AGGGCTTCAAGGACTTACCC | *L. monocytogenes* serovars 1/2a, 3a, 1/2c and 3c | 53 | 691 | (Doumith et al., 2004) |
|  | ACGATTTCTGCTTGCCATTC |  |  |  |  |
| *lmo1118* | AGGGGTCTTAAATCCTGGAA | *L. monocytogenes* serovars 1/2c and 3c | 53 | 906 |  |
|  | CGGCTTGTTCGGCATACTTA |  |  |  |  |
| *ORF2819* | AGCAAAATGCCAAAACTCGT | *L. monocytogenes* serovars 1/2b, 3b, 4b, 4d, 4e and 7 | 53 | 471 |  |
|  | CATCACTAAAGCCTCCCATTG |  |  |  |  |
| *ORF2110* | AGTGGACAATTGATTGGTGAA | *L. monocytogenes* serovars 4b, 4d and 4e | 53 | 597 |  |
|  | CATCCATCCCTTACTTTGGAC |  |  |  |  |
| *prs* | GCTGAAGAGATTGCGAAAGAAG | All *Listeria* species | 53 | 370 |  |
|  | CAAAGAAACCTTGGATTTGCGG |  |  |  |  |
|  | TTTATCCGTACTGAAATTCC |  |  |  |  |
| *prfA* | CTGTTGGAGCTCTTCTTGGTGAAGCAATCG | *L. monocytogenes* | 60 | 1060 | (Notermans et al., 1991) |
|  | AGCAACCTCGGTACCATATACTAACTC |  |  |  |  |
| *mpl* | ATAGCTTTTCAGGCTCATTTCA | *L. monocytogenes* | 60 | 1184 | (Chen et al., 2019) |
|  | ATAGCTTTTCAGGCTCATTTCA |  |  |  |  |
| *plcA* | CTGCTTGAGCGTTCATGTCTCATCCCCC | *L. monocytogenes* | 60 | 1484 | (Notermans et al., 1991) |
|  | CATGGGTTTCACTCTCCTTCTAC |  |  |  |  |
| *inlB* | GATATTGTGCCACTTTCAGGT | *L. monocytogenes* | 60 | 367 | (Xu et al., 2009) |
|  | CCTCTTTCAGTGGTTGGGT |  |  |  |  |
| *plcB* | CTGCTTGAGCGTTCATGTCTCATCCCCC | *L. monocytogenes* | 60 | 436 | (Chen et al., 2014) |
|  | CATGGGTTTCACTCTCCTTCTAC |  |  |  |  |
| *hly* | CTTGCAACTGCTCTTTAGTAACAGC | *L. monocytogenes* | 60 | 706 | (Hudson et al., 2010) |
|  | ACAAGCTGCACCTGTTGCAG |  |  |  |  |
| *iap* | ACAAGCTGCACCTGTTGCAG | *L. monocytogenes* | 60 | 131 | (Furrer et al., 1991) |
|  | TGACAGCGTGTGTAGTAGCA |  |  |  |  |
| *actA* | CGCCGCGGAAATTAAAAAAAGA | *L. monocytogenes* | 60 | 839 | (MoÂ nica SuaÂrez, 2001) |
|  | ACGAAGGAACCGGGCTGCTAG |  |  |  |  |
| *llsX* | TTATTGCATCAATTGTTCTAGGG | LIPI-3 | 52 | 200 | (Clayton et al., 2011) |
|  | CCCCTATAAACATCATGCTAGTG |  |  |  |  |
| *ptsA* | TCCTTTTTCTTTGTTGCGGA | LIPI-4 | 52 | 450 | (Maury et al., 2016) |
|  | TCTGAAGCTGTACGAAGACA |  |  |  |  |

References

Chen, M., Chen, Y., Wu, Q., Zhang, J., Cheng, J., Li, F., et al. (2019). Genetic characteristics and virulence of *Listeria monocytogenes* isolated from fresh vegetables in China. *BMC Microbiol* 19(1)**,** 119. doi: 10.1186/s12866-019-1488-5.

Chen, M., Wu, Q., Zhang, J., Guo, W., Wu, S., and Yang, X. (2014). Prevalence and contamination patterns of *Listeria monocytogenes* in Flammulina velutipes plants. *Foodborne Pathog Dis* 11(8)**,** 620-627. doi: 10.1089/fpd.2013.1727.

Clayton, E.M., Hill, C., Cotter, P.D., and Ross, R.P. (2011). Real-time PCR assay to differentiate Listeriolysin S-positive and -negative strains of *Listeria monocytogenes*. *Appl Environ Microbiol* 77(1)**,** 163-171. doi: 10.1128/AEM.01673-10.

Doumith, M., Buchrieser, C., Glaser, P., Jacquet, C., and Martin, P. (2004). Differentiation of the Major *Listeria monocytogenes* Serovars by Multiplex PCR. *Journal of Clinical Microbiology* 42(8)**,** 3819-3822. doi: 10.1128/jcm.42.8.3819-3822.2004.

Furrer, B., Candrian, U., Hoefelein, C., and Luethy, J. (1991). Detection and identification of *Listeria monocytogenes* in cooked sausage products and in milk by in vitro amplification of haemolysin gene fragments. *J Appl Bacteriol* 70(5)**,** 372-379.

Hudson, J.A., Lake, R.J., Savill, M.G., Scholes, P., and Mccormick, R.E. (2010). Rapid detection of *Listeria monocytogenes* in ham samples using immunomagnetic separation followed by polymerase chain reaction. *Journal of Applied Microbiology* 90(4)**,** 614-621.

Maury, M.M., Tsai, Y.H., Charlier, C., Touchon, M., Chenal-Francisque, V., Leclercq, A., et al. (2016). Uncovering *Listeria monocytogenes* hypervirulence by harnessing its biodiversity. *Nat Genet* 48(3)**,** 308-313. doi: 10.1038/ng.3501.

MoÂ nica SuaÂrez, B.G.-Z., Yolanda Vega, Isabel Chico-Calero, JoseÂ-A. VaÂzquez-Boland (2001). A role for ActA in epithelial cell invasion by *Listeria monocytogenes*. *Cellular Microbiology* 3(12)**,** 12.

Notermans, S.H., Dufrenne, J., Leimeisterwächter, M., Domann, E., and Chakraborty, T. (1991). Phosphatidylinositol-specific phospholipase C activity as a marker to distinguish between pathogenic and nonpathogenic *Listeria* species. *Appl Environ Microbiol* 57(9)**,** 2666-2670.

Xu, X, Wu, Q, Zhang, J.M., Deng, M.Q., and Zhou, Y.H. (2009). Studies on specific detection of *Listeria monocytogenes* in foods by duplex PCR. *Chin J Health Lab Technol* 19**,** 1199-1201.
