# Supplementary material for: Heterogeneity, Characteristics, and Public Health Implications of Listeria monocytogenes in Ready-to-Eat Foods and Pasteurized Milk in China
Source: Front Microbiol. 2020 Apr 15;11:642. doi: 10.3389/fmicb.2020.00642 (PMC7174501; doi:10.3389/fmicb.2020.00642)
Supplement: Supplementary file 2 [file Table_2.DOCX]

Table S2 Primers used for multilocus sequence typing analysis

| Primer | Sequences (5’→3’) | Length (bp) | Tm (°C) |
| --- | --- | --- | --- |
| *abcZoF* | **GTTTTCCCAGTCACGACGTTGTA**TCGCTGCTGCCACTTTTATCCA | 537 | 52 |
| *abcZoR* | **TTGTGAGCGGATAACAATTT**CTCAAGGTCGCCGTTTAGAG |  |  |
| *bglAoF* | **GTTTTCCCAGTCACGACGTTGTA**GCCGACTTTTTATGGGGTGGAG | 399 | 45 |
| *bglAoR* | **TTGTGAGCGGATAACAATTT**CCGATTAAATACGGTGCGGACATA |  |  |
| *catoF* | **GTTTTCCCAGTCACGACGTTGTA**ATTGGCGCATTTTGATAGAGA | 486 | 52 |
| *catoR* | **TTGTGAGCGGATAACAATTT**CAGATTGACGATTCCTGCTTTTG |  |  |
| *dapEoF* | **GTTTTCCCAGTCACGACGTTGTA**CGACTAATGGGCATGAAGAACAAG | 462 | 52 |
| *dapEoR* | **TTGTGAGCGGATAACAATTT**CATCGAACTATGGGCATTTTTACC |  |  |
| *datoF* | **GTTTTCCCAGTCACGACGTTGTA**GAAAGAGAAGATGCCACAGTTGA | 471 | 52 |
| *datoR* | **TTGTGAGCGGATAACAATTT**CTGCGTCCATAATACACCATCTTT |  |  |
| *ldhoF* | **GTTTTCCCAGTCACGACGTTGTA**GTATGATTGACATAGATAAAGA | 453 | 52 |
| *ldhoR* | **TTGTGAGCGGATAACAATTTC**TATAAATGTCGTTCATACCAT |  |  |
| *lhkAoF* | **GTTTTCCCAGTCACGACGTTGTA**AGAATGCCAACGACGAAACC | 480 | 52 |
| *lhkAoR* | **TTGTGAGCGGATAACAATTT**CTGGGAAACATCAGCAATAAAC |  |  |
| *LhkA*-F3 | GCAAGTTTTGAATACGTATCAGCG (Lineage 3) | 480 | 52 |
| *LhkA*-R2 | TACGCATTTCATGAGAAACATCAG (Lineage 3) |  |  |
