# Supplementary material for: Heterogeneity, Characteristics, and Public Health Implications of Listeria monocytogenes in Ready-to-Eat Foods and Pasteurized Milk in China
Source: Front Microbiol. 2020 Apr 15;11:642. doi: 10.3389/fmicb.2020.00642 (PMC7174501; doi:10.3389/fmicb.2020.00642)
Supplement: Supplementary file 3 [file Table_3.docx]

Table S3 Antimicrobial susceptibilities of *Listeria monocytogenes* isolates from ready-to-eat foods and pasteurized milk

| Class of antibiotics | Antibiotics | Susceptible | Intermediate | Resistance | Susceptible (%) | Immediate (%) | Resistance (%) |
| --- | --- | --- | --- | --- | --- | --- | --- |
| Aminoglycosides | Kanamycin | ≥18 | 14-17 | ≤13 | 48 (100.0) | 0 (0) | 0 (0) |
|  | Gentamicin | ≥15 | 13-14 | ≤12 | 48 (100.0) | 0 (0) | 0 (0) |
| Quinolones | Ciprofloxacin | ≥21 | 16-20 | ≤15 | 37 (77.1) | 11 (22.9) | 0 (0) |
|  | Levofloxacin | ≥19 | 16-18 | ≤15 | 47 (97.9) | 1 (2.1) | 0 (0) |
|  | Ofloxacin | ≥16 | 13-15 | ≤12 | 46 (95.8) | 2 (4.2) | 0 (0) |
| Potentiated sulfonamide | Sulfamethoxazole with trimethoprim | ≥16 | 11-15 | ≤10 | 48 (100.0) | 0 (0) | 0 (0) |
| Aminoglycosides | Streptomycin | ≥15 | 12-14 | ≤11 | 19 (39.6) | 0 (0) | 29 (60.4) |
| Nitrofurans | Rifampin | ≥20 | 17-19 | ≤16 | 41 (85.4) | 7 (14.6) | 0 (0) |
| Tetracyclines | Doxycycline | ≥16 | 13-15 | ≤12 | 48 (100.0) | 0 (0) | 0 (0) |
| Chloramphenicols | Chloramphenicol | ≥18 | 13-17 | ≤12 | 46 (95.8) | 2 (4.2) | 0 (0) |
| Macrolides | Erythromycin | ≥23 | 14-22 | ≤13 | 47 (97.9) | 1 (2.1) | 0 (0) |
| Tetracyclines | Tetracycline | ≥19 | 15-18 | ≤14 | 48 (100.0) | 0 (0) | 0 (0) |
| Novel β-Lactam | Meropenem | ≥16 | 14-15 | ≤13 | 48 (100.0) | 0 (0) | 0 (0) |
| polypeptide | Vancomycin | ≥17 | 15-16 | ≤14 | 48 (100.0) | 0 (0) | 0 (0) |
| Oxazolidone | Linezolid | ≥21 | - | ≤20 | 48 (100.0) | 0 (0) | 0 (0) |
| β-Lactam inhibitors | Amoxicillin/clavulanic acid | ≥20 | - | ≤19 | 48 (100.0) | 0 (0) | 0 (0) |
| β-Lactam inhibitors | Sulbactam/ampicillin | ≥15 | 12-14 | ≤11 | 48 (100.0) | 0 (0) | 0 (0) |
| β-Lactam | Ampicillin | ＜2 μg/mL | - | ≥2 μg/mL | 48 (100.0) | 0 (0) | 0 (0) |
| β-Lactam | Penicillin | ＜2 μg/mL | - | ≥2 μg/mL | 48 (100.0) | 0 (0) | 0 (0) |
